# Association of *Mycobacterium africanum* Infection with Slower Disease Progression Compared with *Mycobacterium tuberculosis* in Malian Patients with Tuberculosis

**DOI:** 10.4269/ajtmh.19-0264

**Published:** 2019-11-11

**Authors:** Bocar Baya, Bassirou Diarra, Seydou Diabate, Bourahima Kone, Drissa Goita, Yeya dit Sadio Sarro, Keira Cohen, Jane L. Holl, Chad J. Achenbach, Mohamed Tolofoudie, Antieme Combo Georges Togo, Moumine Sanogo, Amadou Kone, Ousmane Kodio, Djeneba Dabitao, Nadie Coulibaly, Sophia Siddiqui, Samba Diop, William Bishai, Sounkalo Dao, Seydou Doumbia, Robert Leo Murphy, Souleymane Diallo, Mamoudou Maiga

**Affiliations:** 1 University Clinical Research Center (UCRC)–SEREFO Laboratory-University of Sciences, Techniques and Technologies of Bamako (USTTB), Bamako, Mali; 2 Johns Hopkins University School of Medicine, Baltimore, Maryland; 3 Northwestern University, Chicago, Illinois; 4 National Institutes of Allergic and Infectious Diseases (NIAID), Rockville, Maryland

## Abstract

*Mycobacterium africanum* (MAF) is known to endemically cause up to 40–50% of all pulmonary TB in West Africa. The aim of this study was to compare MAF with *Mycobacterium tuberculosis* (MTB) with regard to time from symptom onset to TB diagnosis, and clinical and radiological characteristics. A cross-sectional study was conducted in Bamako, Mali, between August 2014 and July 2016. Seventy-seven newly diagnosed pulmonary TB patients who were naive to treatment were enrolled at Mali’s University Clinical Research Center. Sputum cultures were performed to confirm the diagnosis and spoligotyping to identify the mycobacterial strain. Univariate and multivariate analyses were used to identify factors associated with disease progression. Overall, the frequency of female patients was 25% in MAF infection and only 10.0% in MTB infection (OR = 2.9), and MAF was more represented in patients aged ≥ 30 years (57.1% versus 36.7% [OR = 2.3]). More MAF- than MTB-infected patients had a history of a prior TB contact (32.1% versus 14.3% [OR = 2.8]). The mean duration between cough onset and TB diagnosis was 111 days (∼3.7 months) for MAF and 72 days (∼2.4 months) for MTB (*P* = 0.007). In a multivariate regression, weight loss (body mass index [BMI] < 18.5 kg/m^2^) and cough duration (> 4 months) were strongly associated with MAF infection (OR = 5.20 [1.49–18.26], *P* = 0.010, and 4.74 [1.2–18.58], *P* = 0.02), respectively. Our data show that MAF infection was significantly associated with lower BMI and a longer time between symptom onset and TB diagnosis than MTB. This supports the concept that MAF infection may have slower disease progression and less severe cough symptoms than MTB.

## INTRODUCTION

*Mycobacterium africanum* (MAF), first described in Senegal, has two distinct phylogenic lineages (5 and 6). *Mycobacterium africanum* differs from *Mycobacterium tuberculosis* (MTB) because of the absence of certain regions of difference (RD), with MAF-1 lacking RD9 and MAF-2 lacking RD7 through 10.^1^ *Mycobacterium africanum* is known to endemically cause up to 40–50% of all pulmonary TB in West Africa.^1–3^ Many countries geographically, culturally, and/or economically linked to Senegal also report the presence of these lineages. However, prevalence of MAF varies among other West African countries, ranging from 2.7% in Cameroon to 38% in Gambia; 20% in clinical samples in Burkina Faso, 5% in Guinea, Conakry, and 22% in Ivory Coast; and 12.6% with MAF1 and 9.2% with MAF2 in Ghana.^2,4–8^

In Mali, three laboratory-based studies reported on MAF prevalence: 29.7% (1.7% MAF-1, 28% MAF-2) in TB cases in 2010, an average of 17.9% (17.0 MAF-1 and 0.9 MAF-2) over a 10-year period in 2017, and 24.3% (22.9 MAF1 and 1.4% MAF2) in 2018.^5,9,10^ Studies in other continents support the concept that MAF is geographically restricted to West Africa. For example, an epidemiological study of MAF TB cases in the United States found an adjusted odds of 253.8-fold higher risk of MAF in people of West African origin than MTB*.*^11^ Another study confirmed that MAF emergence in higher income countries is due to immigration from West Africa.^12^ Clinical and microbiological studies have shown that MAF grows more slowly and causes less severe tuberculosis.^13–15^ De Jong et al.,^5^ in 2010, reported that lower body mass index (BMI) and moderate lung damage on chest X-ray were associated with MAF West African type 2 compared with MTB. *Mycobacterium africanum* isolates were 2.52-fold less likely to produce a positive sputum smear result than MTB cases, suggesting lower lung bacterial loads.^16^ In addition to its slow growth in culture, MAF has low bacterial virulence that probably delays disease progression during infection. Furthermore, host tolerance, which may result in a longer time period between the onset of cough symptoms and diagnosis, may also play a role.

The aim of this study was to compare the two most common strains in Mali, *M. africanum* lineage 5 and/or 6 (MAF), and *M. tuberculosis* lineage 4 (Latin American [LAM] 10 and T1 family) (MTB), by assessing the time from onset of the first symptom to diagnosis (time to diagnosis) and the clinical and radiological aspects of disease severity.

Improved knowledge of the differences in the clinical presentation of MAF compared with MTB may help clinicians in the early diagnosis of MAF tuberculosis at the stage of mild cough without remarkable weight loss or fever to limit the extension of pulmonary lesions or deterioration of the physical condition.

## METHODS

### Study design and setting.

A cross-sectional study was conducted between August 2014 and July 2016. Participants were recruited and enrolled at the University Clinical Research Center (SEREFO-UCRC) clinic at the Department of Respiratory and TB Diseases of the Point-G University Teaching Hospital, Bamako, Mali. All laboratory tests were performed at the HIV/TB Research and Training BSL-3 Laboratories of the SEREFO-UCRC at the University of Sciences, Techniques and Technologies of Bamako (USTTB) in Mali.

### Participants and mycobacterial strains.

We consecutively enrolled patients, newly diagnosed with pulmonary TB, but naive to TB treatment, who consented to participate and accepted to be tested for HIV. Patients with a negative sputum mycobacterial culture or diagnosed with nontuberculous mycobacteria were further excluded, in addition to patients who were HIV positive. Patients infected with HIV were excluded because of their very small number in our sample. All patients underwent a standardized interview about onset of cough symptoms and chronology of cough, and a physical examination. After culture, MTB subspecies isolation and identification were performed by spoligotyping. We chose to compare data from MAF and MTB lineage 4 (*T1* and *LAM-10*) cases because these three strains had been the most prevalent in Mali during the prior 10 years.

### Data collection.

Sociodemographic data, including age, gender, ethnicity, marital status, occupation, and residence, were collected, as well as information about household contact with a previous TB patient and smoking status. A medical history, including date of onset of cough symptoms and chronology of cough, was obtained, and a physical examination was conducted, including BMI and presence and size of any Bacillus Calmette-Guérin (BCG) scar. A blood sample was collected for HIV testing and complete blood count (CBC). Two early morning sputum samples were collected on 2 consecutive days for direct microscopy after concentration, mycobacterial culture, and species identification. Chest X-ray results were recorded (lesion localization to one or both lungs and presence of cavities). All patients then received the standard, 6-month, first-line TB regimen, according to the Mali National TB Program Protocol, which includes 2 months of rifampicin (R), isoniazid (I), pyrazinamide, (P) and ethambutol (E), followed by 4 months of R+H (2RHZE/4RH), after inclusion in the study.

### Laboratory tests.

#### Sputum microscopy, mycobacterial cultures, and subspecies identification.

Sputum specimens were first decontaminated and digested using the standard N-acetyl L-cysteine/4% NaOH solution and concentrated by centrifugation (4,500 rpm). The pellets were used for smear microscopy staining using the auramine–rhodamine method. Both liquid (Mycobacteria Growth Incubator Tube [BBL^™^ MGIT^™^ Becton Dickinson, Sparks, MD]) and solid (Middlebrook 7H11 agar and selective 7H11 agar, Lenexa, KS) media were inoculated. Speciation of positive acid-fast bacilli cultures was performed with nucleic acid probes (AccuProbe^®^ GenProbe, San Diego, CA). *Mycobacterium tuberculosis* complex (MTBc) isolates were spoligotyped using a commercially available kit (Isogen Life Science, De Meern, The Netherlands) to differentiate MTB and MAF species and subspecies.

### HIV serology test and CBC.

HIV testing was performed using *Determine*^®^ (Abbott Laboratories, Matsudo-Shi, Chiba, Japan) followed by an HIV ELISA test (Genscreen Ag-Ac UltraHIV-1/2 version 2 Assay, Bio-Rad Laboratories, Marnes, France). All positive ELISA tests were further confirmed by the Western blot assay (New Lav Blot I and II, Bio-Rad Laboratories). The CBC was performed using a coulter counter (CELL-DYN Ruby; Abbott^®^, Chicago, IL).

### Data analysis.

Data from the first 77 participants diagnosed with pulmonary tuberculosis, including 28 cases of MAF-infected and 49 MTB-infected patients from an ongoing study, “Host–Pathogen Interactions in a Failing Global Lineage of MTBC: *M. africanum* (R01 AI110386, PI: Souleymane Diallo),” were analyzed. Data were deidentified, encrypted, and analyzed with SPSS version 25 for Windows. Frequencies were calculated for all descriptive variables. For independent samples, a *t-*test was used to compare means between the two groups, and a Fisher exact test was used to compare percentages; the level of significance was set at *P* < 0.05. In univariate and multivariate models, we assessed factors associated with MAF, including age, gender, history of household TB contact, smoking status, BMI, BCG scar, cough duration before TB diagnosis, and smear bacterial load. Body mass index was used as a surrogate marker of weight loss because we could not measure actual weight loss. Multivariate logistic regression was performed with MAF and MTB as the dependent variables. In addition to bacterial load (*P =* 0.45), all associated factors with a *P*-value less or equal to 0.1 (age, gender, household TB contact, smoking, BMI, and cough duration) were included in the model. Differences were considered significant if the *P*-value was less than 5% (*P* < 0.05), and a 95% CI was used to determine the precision of the difference.

### Ethics considerations.

The study was approved by both the Ethics Committee of the Faculty of Medicine, Pharmacy, and Odontostomatology of the USTTB and Northwestern University Institutional Review Board. Written informed consent was obtained from each study participant before any data collection. Precounseling was provided before HIV testing, and all HIV-positive patients received a post-counseling session and were then referred to an HIV clinic physician.

## RESULTS

### Patient characteristics.

Data from 77 HIV-uninfected, culture-confirmed pulmonary TB patients, who were treatment naïve, were included in the study. As shown in Table 1, 65 (84.4%) patients were male; 43 (55.8%) were aged between 18 and 30 years. Twenty-eight (28) were infected with MAF and 49 with MTB lineage 4 (LAM 10 or T1 family). Chest X-ray results were available for 52 patients; of these, 30 (57.7%) had unilateral infiltrates and 11 (21.2%) had a lung cavity. The mean period between symptom onset and diagnosis was 86.09 ± 61.9 days (7–240 days). In 18 patients (23.4%), TB was diagnosed after 120 days (4 months) (see Table 1).

**Table 1:** Characteristics of study participants at enrollment

Characteristics, *N* = 77	All patients, *n* (%)	*Mycobacterium africanum*, *n* (%)	*Mycobacterium tuberculosis* lineage 4, *n* (%)	Chi^2^ (*P*-value)
Gender				
Female	12 (15.6)	7 (25.0)	5 (10.2)	2.9 (0.08)
Male	65 (84.4)	21 (75.0)	44 (89.8)	
Age (years)				
18–30	43 (55.8)	12 (42.9)	31 (63.3)	3.0 (0.08)
31–62	34 (44.2)	16 (57.1)	18 (36.7)	
Ethnicity				
Bambara	26 (33.8)	5 (17.9)	21 (42.9)	4.9 (0.02)
Peulh	13 (16.9)	7 (25.0)	6 (12.2)	
Malinke	11 (14.3)	6 (21.4)	5 (10.2)	
Soninke	9 (11.7)	2 (07.1)	7 (14.3)	
Others	18 (23.4)	8 (28.6)	10 (20.4)	
Marital status				
Married	40 (51.9)	16 (57.1)	24 (49.0)	0.4 (0.5)
Single	37 (48.1)	12 (42.9)	25 (51.0)	
Smoking				
Yes	22 (28.6)	4 (14.3)	18 (36.7)	4.4 (0.03)
No	55 (71.4)	24 (85.7)	31 (63.3)	
Household contact				
Yes	16 (20.8)	9 (32.1)	7 (14.3)	3.4 (0.06)
No	61 (79.2)	19 (67.9)	42 (85.7)	
Hemoptysis				
Yes	9 (11.7)	3 (10.7)	6 (12.2)	0.04 (0.8)
No	68 (88.3)	25 (89.3)	43 (87.8)	
Night sweat				
Yes	38 (49.4)	14 (50.0)	24 (49.0)	0.007 (0.9)
No	39 (50.6)	14 (50.0)	25 (51.0)	
BCG				
Yes	64 (83.1)	24 (85.7)	40 (81.6)	0.2 (0.6)
No	13 (16.9)	4 (14.3)	9 (18.4)	
Fever at inclusion				
Yes (*T*° ≥ 38.0)	20 (26.0)	7 (25.0)	13 (26.5)	0.02 (0.9)
No (*T*° < 38.0)	57 (74.0)	21 (75.0)	36 (73.5)	
Hemoglobin level (g/dL)				
≤ 10.5	30 (39.0)	10 (35.7)	20 (40.8)	0.2 (0.6)
> 10.5	47 (61.0)	18 (64.3)	29 (59.2)	
Body mass index				
< 18.49	41 (53.2)	20 (71.4)	21 (42.9)	5.8 (0.01)
≥ 18.50	36 (46.8)	8 (28.6)	28 (57.1)	
Cough duration (days)				
> 120 (4 months)	18 (23.4)	11 (39.3)	7 (14.3)	6.2 (0.01)
≤ 120	59 (76.6)	17 (60.7)	42 (85.7)	
Sputum smear results				
Acid-fast bacilli 3+	69 (89.6)	24 (85.7)	45 (91.8)	0.7 (0.4)
Acid-fast bacilli < 3+	8 (10.4)	4 (14.3)	4 (08.2)	
Unilateral infiltrate (*N* = 52)				
Yes	30 (57.7)	10 (50.5)	20 (62.5)	0.8 (0.3)
No	22 (42.31)	10 (50.5)	12 (37.5)	
Pulmonary cavity (*N* = 52)				
Yes	11 (21.2)	2 (10.0)	9 (28.1)	2.4 (0.1)
No	41 (78.8)	18 (90.0)	23 (71.9)	

### Differences between MAF- and MTB lineage 4–infected patients.

**Figure 1.**
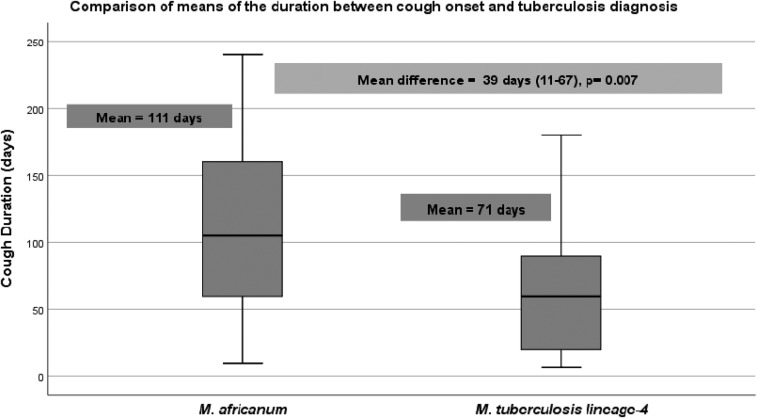
Comparing average period from first cough onset and tuberculosis diagnosis.

*Mycobacterium africanum* infection was higher in female patients (25% versus 10.0%, OR = 2.9) and in patients aged > 30 years (57.1% versus 36.7%, OR = 2.3). However, these findings were not statistically significant at *P* = 0.10 and 0.09, respectively. *Mycobacterium africanum*–infected patients were significantly less likely to be smokers than MTB-infected patients (14.3% versus 36.7%, OR = 0.3, *P* = 0.040). The history of TB contact was not significantly higher among patients with MAF (32.1% versus 14.3%, OR = 2.8, *P* = 0.08), and the presence of a BCG vaccination scar did not differ (OR = 1.4, *P* = 0.76). A statistically significant difference in mean time between symptom onset and TB diagnosis between MAF (111 days [3.7 months]) and MTB (72 days [2.4 months]) was found (*P* = 0.007) (Figure 1). There were no statistically significant differences in type of symptoms at onset between the two groups, including hemoptysis (OR = 0.8, *P* = 1.0), night sweats (OR = 1.04, *P* = 1.0), or fever at admission (OR = 1.23, *P* = 0.78). There were no significant differences in positive sputum smear microscopy (50 versus 53, *P* = 0.45) or hemoglobin level ≤ 10.5 g/dL (OR = 0.8, *P* = 0.80). Weight loss (BMI < 18.5 kg/m^2^) was significantly associated with MAF infection (OR = 3.33, *P* = 0.019). Unilateral infiltrates and lung cavity on chest X-ray were not associated with neither MAF nor MTB (OR = 0.60, *P* = 0.40, and OR = 0.28, *P* = 0.17), respectively (Table 2). When comparing patients diagnosed ≥ 4 months after onset of cough symptoms, MAF-infected patients were more likely to experience a longer time to diagnosis than MTB lineage 4 (OR = 3.88, *P* = 0.023) patients. In the multivariate regression, weight loss (BMI < 18.5 kg/m^2^) and cough duration (> 4 months) were strongly associated with MAF (aOR = 5.20 [1.49–18.26], *P* = 0.010, and 4.74 [1.2–18.58], *P* = 0.026), respectively, after adjusting for chest X-ray results were missing for 25 patients, including 8/28 (28.6%) for MAF and 17/49 (34.7%) for MTB-infected patients’ age, gender, smoking status, history of household TB contact, and smear bacterial load (Table 2).

**Table 2:** Factors associated with *Mycobacterium africanum* and *Mycobacterium tuberculosis* lineage 4 at inclusion

Characteristics	Unadjusted OR	95% CI	*P*-value	Adjusted OR	95% CI	*P*-value
Gender						
Male	1	–	–	1	–	0.10
Female	2.9	(0.8–10.3)	0.10	4.2	(0.8–23.6)	
Age range (years)						
18–30	1	–	–	1	–	0.13
31–62	2.3	(0.9–5.9)	0.09	2.4	(0.7–7.5)	
Marital status						
Single	1	–	–	–	–	
Married	1.4	(0.5–3.5)	0.63	–	–	
Smoking						
No	1	–	–	1	–	0.16
Yes	0.3	(0.09–0.9)	0.040*	0.36	(0.09–1.5)	
Household contact						
No	1	–	–	–	–	0.32
Yes	2.8	(0.9–8.8)	0.082	1.95	(0.5–7.4)	
Hemoptysis						
No	1	–	–	–	–	
Yes	0.8	(0.2–3.7)	1.0	–	–	
Night sweat						
No	–	–	–	–	–	
Yes	1.04	(0.4–2.6)	1.0			
Fever at inclusion						
No (*T*° < 38.0)	1	–	–	–	–	
Yes (*T*° ≥ 38.0)	0.9	(0.3–2.7)	0.8			
BCG vaccinated						
No	1	–	–	–		
Yes	1.4	(0.4–4.9)	0.76			
Smear results at baseline						
AFB seen < 3+	1	–	–	1	–	0.76
AFB seen 3+	0.53	(0.1–2.3)	0.45	0.74	(0.1–4.9)	
Hemoglobin level (g/dL)						
> 10.5	1	–	–	–	–	
≤ 10.5	0.80	(0.3–2.1)	0.80	–	–	
Body mass index						
≥ 18.50	1	–	–	1	–	0.010*
< 18.49	3.33	(1.2–9.0)	0.019*	5.2	(1.5–18.3)	
Cough duration (days)						
≤ 120 (4 months)	1	–	–	1	–	0.026*
> 120	3.88	(1.3–11.7)	0.023*	4.74	(1.2–18.6)	
Unilateral infiltrate						
No	1	–	–	–	–	
Yes	0.60	(0.2–1.9)	0.40	–	–	
Pulmonary cavity (*N* = 52)						
No	1	–	–	–	–	
Yes	0.28	(0.05–1.5)	0.17	–	–	

AFB = acid-fast-Bacilli.

* *P*-value significant at 95% confidence interval.

## DISCUSSION

Considering the geographical restriction of MAF to West Africa, as shown in several prior studies,^2,4–8^ MTB nevertheless predominates in West Africa. A 2017 study on spoligotyping classified MAF infection as the third leading cause of TB in Mali.^10^ However, the MTB strain is progressively becoming the most prevalent. In this article, we describe the clinical presentation of MAF- compared with MTB-infected patients in Mali. The general demographic characteristics of our study population are similar to the De Jong et al.^5^ findings in Gambia; however, fewer MAF patients in Mali were aged ≥ 65 years (OR: 0.2, 95% CI: 0.1–0.5) than MAF patients in the United States,^11^ which is likely explained by the younger age of most patients in Mali than U.S. patients. Smoking has been reported as a risk factor, in general, for TB infection, progression to active disease, and poor treatment outcomes.^17^ Asante-Poku et al.^18^ reported a 2-fold greater risk for MAF patients who smoke than MTB patients. Although our study found that MAF-infected patients were less likely to be smokers than MTB lineage 4–infected patients (OR = 0.3, 95% CI: 0.09–0.9, *P* = 0.040) in the bivariate analysis, after adjusting in the multivariate regression model, the difference was not significant. Tuberculosis is known to be associated with immune suppression, and indeed, patients experienced weight loss (BMI < 18.5 kg/m^2^) as an independent associated factor for MAF infection, which supports the findings of several other studies.^1,5^ The results of our study suggest that MAF has low virulence or slower multiplication, affecting its ability to cause severe symptoms early in the progression of infection, which may explain why patients are often first seen after having significant weight loss. In this study, patients with MAF infection had a 2-fold higher risk of having a household TB contact, but the association was not statistically significant, which is aligned with the findings of De Jong et al.^19^ who found that household contacts whose index case had MTB were more likely to develop TB disease than contacts of MAF-infected patients. In this study, bacterial load, although different, was not statistically significant between the two groups (OR = 0.53; *P* = 0.45), as previously noted by Tientcheu et al.^20^ As expected, all 52 patients had lung abnormalities on chest X-ray, but MAF-infected patients showed lower odds of having unilateral infiltrates and lung cavities (OR = 0.60, *P* = 0.40, and OR = 0.28, *P* = 0.17), respectively. Although the associations were not significant, a similar observation was reported in a U.S. study by Sharma (OR 0.5, 95% CI: 0.4–0.7).^11^ However, De Jong et al.^1^ reported similar radiological lesions between groups in Gambia.

These data show that there is a long period between onset of cough symptoms and formal diagnosis in the MAF-infected group. However, association with time to diagnosis remains strong even after adjusting for age, gender, bacterial load, smoking, and BMI. Delay in diagnosis may be related to factors beyond strain virulence, including patients’ health beliefs, socioeconomic status, and education levels. Symptom severity is certainly another important factor that influences patients’ decision to seek health care. De Jong et al.^19^ showed that MAF transmission to household members and disease progression suggest a rate of transmission similar to MTB; however, infection progression to active TB is slower in MAF. A recent meta-analysis comparing strain transmission of West African MAF showed a relative risk of 0.61, 95% CI: 0.43–0.86, *P* = 0.01, compared with the Euro-American lineage 4 strain.^21^ Taken together, these data support an association of MAF infection with host specificity, including age, severity of cough symptoms, and slow progression to disease, all of which support a relative lack of virulence of MAF compared with MTB. On the other hand, the strain seems virulent enough to cause disease at least in West African populations and to maintain similar bacterial loads in both types of strains. We have shown that MAF does not lack virulence genes compared with MTB but has lineage-specific mutations, such as mutations in cell wall structure, secretion systems, and cofactor biosynthesis.^22^ We hypothesize that immunogenetic factors, such as HLA types and host microbiome, may be contributing factors that explain its geographical restriction to the West African population.^23^ Many studies are currently ongoing in West Africa to address the question of slower progression of MAF infection that could be exploited for new vaccine strategies.

### Study limitations.

This pilot study revealed many important differences of MAF infection, some known and others new, especially in the Malian population. Some associations trended toward significance but were not statistically significant. Chest X-ray results were missing for 25 patients, including 8/28 (28.6%) for MAF-infected and 17/49 (34.7%) for MTB-infected patients; the exclusion of HIV-infected patients and the small sample size may have prevented the discovery of certain associations or the strength of their relationships. A larger sample for a confirmatory study is necessary. In addition, future studies should include other TB lineages for complete analysis. Nevertheless, this study contributes to understanding MAF disease and sets the stage for future mechanism and population studies that may help to debunk the myth of this disease’s preference for West African populations or the resistance of populations, which may facilitate the design of future TB vaccines.

## CONCLUSION

Our data show that MAF infection was significantly associated with weight loss and a considerably longer period between cough symptom onset and TB diagnosis compared with MTB, suggesting that MAF infection may have slower disease progression and present less severe cough symptoms than MTB. West African hosts may have immunological and/or genetic and/or the microbiota backgrounds that delay the progression of MAF infection compared with MTB. Prospective studies are needed to confirm these findings.
